# Adaptation of Grapevine Flowers to Cold Involves Different Mechanisms Depending on Stress Intensity

**DOI:** 10.1371/journal.pone.0046976

**Published:** 2012-10-10

**Authors:** Mélodie Sawicki, Etienne Jeanson, Vanessa Celiz, Christophe Clément, Cédric Jacquard, Nathalie Vaillant-Gaveau

**Affiliations:** Laboratoire de Stress, Défenses et Reproduction des Plantes, Unité de Recherche Vignes et Vins de Champagne - EA 4707, UFR Sciences Exactes et Naturelles, Université de Reims Champagne-Ardenne, BP 1039, Reims, France; RIKEN Plant Science Center, Japan

## Abstract

Grapevine flower development and fruit set are influenced by cold nights in the vineyard. To investigate the impact of cold stress on carbon metabolism in the inflorescence, we exposed the inflorescences of fruiting cuttings to chilling and freezing temperatures overnight and measured fluctuations in photosynthesis and sugar content. Whatever the temperature, after the stress treatment photosynthesis was modified in the inflorescence, but the nature of the alteration depended on the intensity of the cold stress. At 4°C, photosynthesis in the inflorescence was impaired through non-stomatal limitations, whereas at 0°C it was affected through stomatal limitations. A freezing night (−3°C) severely deregulated photosynthesis in the inflorescence, acting primarily on photosystem II. Cold nights also induced accumulation of sugars. Soluble carbohydrates increased in inflorescences exposed to −3°C, 0°C and 4°C, but starch accumulated only in inflorescences of plants treated at 0 and −3°C. These results suggest that inflorescences are able to cope with cold temperatures by adapting their carbohydrate metabolism using mechanisms that are differentially induced according to stress intensity.

## Introduction

In temperate species, cold stress can result from exposure to temperatures between 0 and 4°C (chilling) [1] or below 0°C (freezing). Both chilling and freezing temperatures adversely affect plant growth and development, constraining spatial expansion and productivity. Cold can trigger adaptive processes or lead to alterations in physiological traits when stress intensity exceeds a certain threshold. Adaptation to cold can include (i) calcium mediated transduction [2], [3] resulting in activation of cold-specific pathways such as *CRT/DREB1*, and induction of the expression of *ICE* and subsequently of *CBF* transcription factors, as well as activation of ABA-related metabolism [4], (ii) accumulation of osmoprotectant molecules such as sugars [5], [6], [7] or amino acids [8], [9] and (iii) post-transcriptional regulation of proteins involved in cold acclimation [10]. When protective mechanisms are not induced at the appropriate time, plant cell structures may suffer cold injuries, particularly a decrease in membrane lipid mobility [11], [12], [13] and a reduction in photosynthesis [14]. Photosynthesis is one of the traits most rapidly affected by cold temperatures [15], as manifested by stomatal closure [16]. The chloroplast is the organelle most severely affected by cold temperatures [17], [18], as revealed by deregulation of the photosynthetic chain [19] and perturbation of key photosynthesis related enzymes [20], [21]. The arrest of plant growth due to cold temperatures also induces feedback inhibition of the whole photosynthetic process [22].

The distribution of sugars between different plant organs is also modified at low temperatures, independently of alterations in photosynthesis. Changes in sugar concentrations are of particular importance, since carbohydrate status modulates and coordinates internal regulators and environmental cues that control growth and development [23], [24]. Most abiotic stresses, including water deficit [25], salt or osmoticum fluctuations [26], [27], and low temperatures [5], have been reported to lead to major alterations in carbohydrate contents.

Reports to date on sugar fluctuations in grapevine during cold acclimation have mainly focused on vegetative organs. Energy-related carbohydrates such as starch that accumulate in buds at low temperatures [28] can provide reserves which may be mobilized during the winter to supply plant cells with osmolyte. Less abundant sugars, putatively involved in signaling, also altered when grapevine plants are exposed to cold. It has been demonstrated that oligosaccharides such as raffinose are accumulated by winter buds and contribute to cold hardiness [28], [29]. More recently, it has been shown that both trehalose and trehalose-6-phosphate fluctuate in plantlets grown *in vitro*, making T6P a putative signalling molecule in cold stress [30].

In grapevine, the correct formation of sexual organs and the success of sexual reproduction are dependent upon sugar supply and may be affected by any form of stress leading to a shortage of carbohydrates [31]. Carbohydrate supply is crucial at key stages of reproductive organ formation, from the initiation of inflorescence up to fruit set [32], [33]. Sexual organs are sensitive to stresses that induce sugar deprivation [34], especially during meiosis [31], [35], [36], [37]. In the grapevine flower, female meiosis coincides with drastic physiological changes in the whole plant. At this time, carbon nutrition switches from mobilization of wood reserves to photosynthesis in the leaves [31], [38], [39]. The grapevine inflorescence also contains chlorophyll from bud burst up until berry ripening, supporting photosynthesis which contributes to the reproductive effort [33], [37], [40], [41]. Perhaps surprisingly, photosynthates produced in the inflorescence during flower development are mainly distributed to vegetative organs [42]. Fluctuations in photosynthesis due to cold stress may participate in inflorescence adaptation to low temperatures.

In regions with a temperate climate, cold nights can occur in late spring at the time of female meiosis in grapevine flowers. Cold stress may therefore disturb carbon metabolism in the inflorescence, leading to ovule abortion and reducing fruit set [43], [44], [45], [46]. Although changes in sugar concentrations have been characterized in vegetative organs following cold treatment [28], limited information is available about fluctuations in carbon metabolism in inflorescences at low temperatures. We therefore investigated the early physiological responses of grapevine inflorescences, particularly variations in photosynthesis and carbohydrate content, following a night at either chilling or freezing temperature.

## Materials and Methods

### Plant material

Experiments were performed on Pinot noir (*Vitis vinifera* L.) fruiting cuttings obtained from grapevine canes according to the improved protocol of Lebon et al. [33]. Cuttings were planted in pots containing a perlite/sand mixture (1/2, v/v) and transferred to a growth chamber at 25°C/19°C (day/night), at a relative humidity of 60% and a 16 h light/8 h dark photoperiod (photosynthetically active radiation, PAR,  = 300 µmol.m^−2^.s^−1^).

Cuttings with four leaves and the inflorescence at female meiosis were placed at 4°C (standard cold temperature), 0°C (freezing point) or −3°C (sub-lethal freezing temperature) for a 12 h night as suggested by Bertamini et al. [21]. Pots were insulated with cardboard and cotton to protect the roots. Control plants were maintained in a growth chamber for 12 h at 19°C.

### Ethics

No specific permits were required for the field studies described. No specific permissions were required for these locations/activities. The location is not privately-owned or protected in any way. The field studies did not involve endangered or protected species.

### Inflorescence gas exchange

The net photosynthesis (Pn), intercellular CO_2_ concentration (C_i_) and stomatal conductance (g_s_) of inflorescences were determined simultaneously with an open gas exchange system (LI-6400, Li-Cor, Lincoln, USA), using equations developed by von Caemmerer and Farquhar [47]. The system was equipped with a 6400-22L Package (6400-22L Lighted Conifer Chamber and 6400-18 RGB Light Source). Air temperature and relative humidity were maintained at 25°C and 30%, respectively. Photosynthetically active radiation was fixed at 1500 µmol.m^−2^.s^−1^. Carbon dioxide concentration was maintained at a constant level of 380 µmol.L^−1^ using a LI-6400-01 CO_2_ injector with a high-pressure liquid CO_2_ cartridge source. The same plants were used for all measurements at time points corresponding to 2, 24, 48, 120 and 192 h after the end of the stress treatment. Gas exchange measurements were performed 3 times per inflorescence. Five plants were used per treatment and time point and three biological replicates were performed (total n = 45).

### Chlorophyll a fluorescence

Photosystem II (PSII) efficiency and excitation energy dissipation in grape inflorescences were examined using modulated fluorescence techniques. Chlorophyll *a* fluorescence was quantified on the flowers and stalk of the same inflorescence with a chlorophyll imaging system (IMAGING-PAM, Walz, Effeltrich, Germany). Inflorescences were dark adapted for 30 min to determine the minimal level of fluorescence (F_0_) and the maximal fluorescence (F_m_) after a saturating flash (1 s, 2500 µmol.m^−2^.s^−1^). Actinic illumination (100 µmol.m^−2^.s^−1^) was applied after fluorescence stabilization. A second saturating flash (2 s) was imposed to determine the maximal fluorescence (F_m_^′^) of a light-adapted inflorescence. Removal of the actinic light and exposure to a short period of far-red light allowed measurement of the zero level of fluorescence (F_0_′). In both dark- and light-adapted states, the fluorescence parameters were calculated according to Schreiber et al. [48] and Genty et al. [49]. In addition, both photochemical (q_P_) and non-photochemical quenching (q_NP_) were calculated according to van Kooten and Snel [50]. Chlorophyll *a* fluorescence measurements were performed on the flowers and stalk of the same inflorescence that was used for gas exchange measurements.

### Carbohydrate analysis

Inflorescences were collected 2, 24 and 48 h after application of the stress treatments, frozen in liquid nitrogen (N_2_) and stored at −80°C until required for determination of sugar content. Three plants were used per treatment with three measurements per time point, and three biological replicates were performed (total n = 27).

#### Extraction

Inflorescences were ground in a mortar with liquid N_2_, and 100 mg of the powder was used to determine sugar concentration. Carbohydrates were extracted in the presence of sodium phosphate buffer (0.1 M, pH 7.5). The extract was centrifuged at 4°C for 15 min at 10000 g. The supernatant was used for soluble sugar determination and the pellet for starch determination. The pellet was suspended in a 1 ml of mixture containing dimethylsulfoxide:hydrochloric acid 8 N (4/1, v/v) and starch was dissolved for 30 min at 60°C with continuous shaking. After cooling, the extract was centrifuged at 20°C for 5 min at 5000 g and the supernatant was stored at −20°C until required.

#### Glucose, fructose and sucrose assay

Soluble sugar analyses were performed using ENZYTEC™ D-glucose/D-fructose/sucrose kits (Germany) according to the manufacturer's instructions. Briefly, D-glucose was phosphorylated and oxidized in the presence of NADP to gluconate-6-phosphate and NADPH, H^+^. The amount of NADPH, H^+^ formed was determined from its absorbance at 340 nm. Fructose was phosphorylated to fructose-6-phosphate by a hexokinase in the presence of ATP. Fructose-6-phosphate was then converted to glucose-6-phosphate by a phosphoglucose isomerase. The glucose-6-phosphate formed was tested as described above and a blank was prepared without phosphoglucose isomerase. Sucrose was hydrolyzed to D-glucose and D-fructose with β-fructosidase. The D-glucose formed was then determined as described above, using a blank without β-fructosidase.

#### Starch assay

Aliquots of 100 µl extract were used to determine starch concentration. Each aliquot was mixed with 100 µl Lugol's iodine solution (0.03% I_2_ and 0.06% Kl in 0.05 N HCl) and placed in the dark. After 15 min, the absorbance was read using a spectrophotometer at 600 nm. A blank was prepared containing the starch solvent (DMSO/HCl, 4/1) instead of extract [31].

### Statistical analysis

All data were analyzed using Student's *t* test and a repeated two-way analysis of variance (ANOVA). An additional one-way ANOVA was used for non-significant parameters to determine whether the values for treated plants were significantly different from those for control plants at each time point.

## Results

### Photosynthesis in the inflorescence is impaired following a cold night

#### Cold at night disturbs inflorescence gas exchange

**Figure 1:**
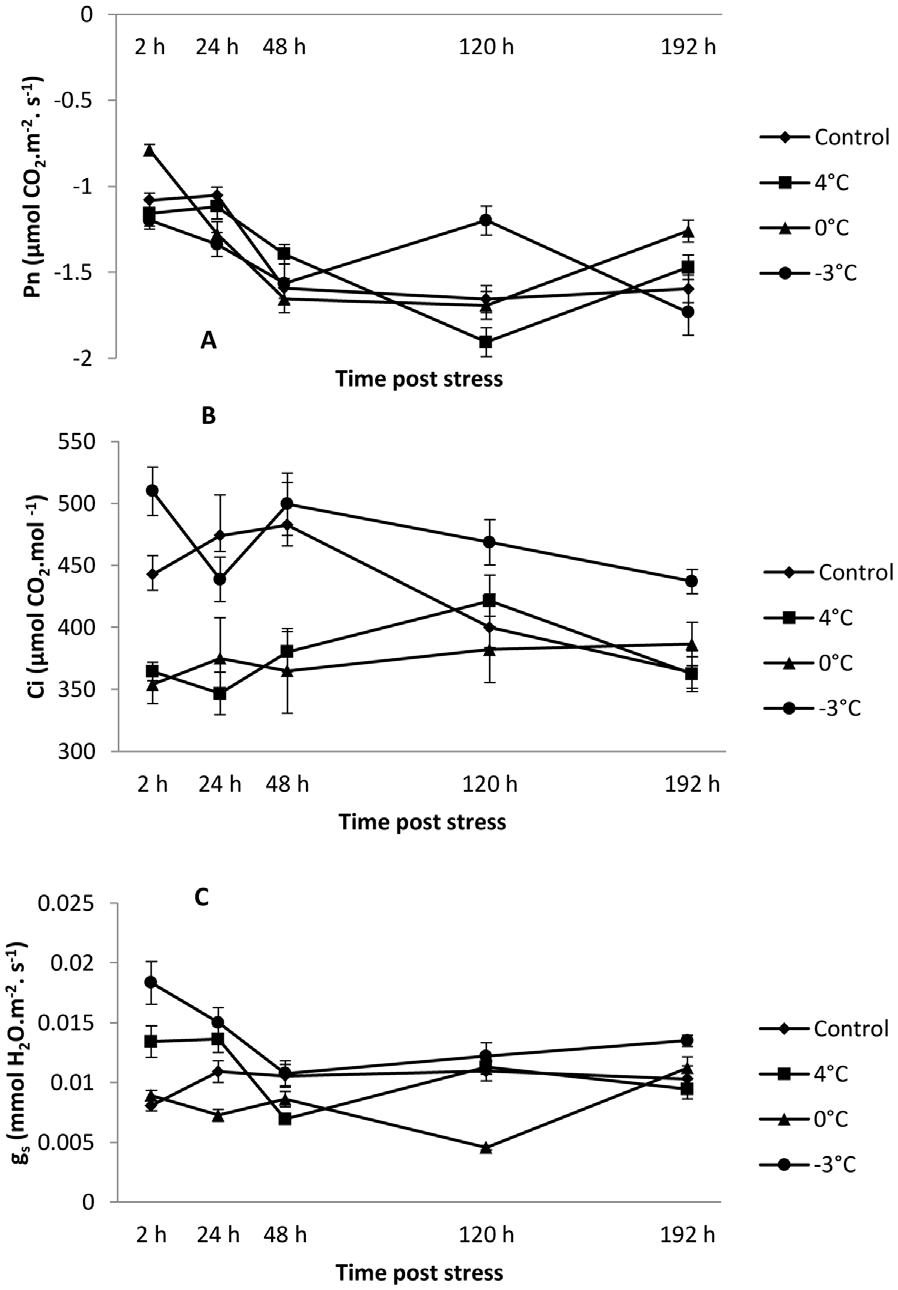
Changes in (A) net photosynthesis (Pn), (B) intercellular CO_2_ concentration (Ci) and (C) stomatal conductance (g_s_) in control and 4°C, 0°C or −3°C-treated inflorescences. Measurements were performed 2, 24, 48, 120 and 192 h after cold treatment. Values are means ± standard errors (n = 45).

Two-way analysis of variance reveals that the night-time temperature has an impact on gas exchange parameters (Table 1). Net photosynthesis (Pn) in the inflorescence was negative in both control and treated plants throughout the whole experiment (Figure 1A). This indicates that the amount of carbon fixed by photosynthesis in this organ was less than the amount produced by respiration. Net photosynthesis was altered after the night treatment as revealed by one-way ANOVA comparing control and treated inflorescences at 4°C, 0°C and −3°C, respectively (Table 2). After a night at 4°C, Pn significantly increased at 48 h (+12%) and decreased by 120 h (−15%) after the end of stress. After 0°C treatment, Pn increased at 2 h (+27%) and 192 h (+21%) but was reduced at 24 h (−21%). In response to a night at −3°C, Pn was reduced at 2 h (−11%) and 24 h (−27%), whereas it increased at 120 h (+28%).

**Table 1:** Results of two-way ANOVA of the impact of night temperature on gas exchange parameters.

Variable	Kinetics	Night treatment	Interaction
**Pn**	***	ns	***
**Ci**	*	***	***
**g_s_**	***	***	***

Significant results at P≤0.05 (*), P≤0.01 (**), P≤0.001 (***) and not significant (ns), respectively.

**Table 2:** Results of one-way ANOVA of the impact of night temperature on net photosynthesis (Pn) and stalk non-photochemical quenching (q_NP_), compared to the control, at each time point. PS, post stress.

Variable	2 h PS	24 h PS	48 h PS	120 h PS	192 h PS
**Pn**					
**4°C**	ns	ns	**	*	ns
**0°C**	***	**	ns	ns	**
**−3°C**	*	***	ns	***	ns
**q_NP_**					
**4°C**	ns	ns	*	ns	ns
**0°C**	ns	ns	*	*	ns
**−3°C**	**	ns	ns	ns	ns

Significant results at P≤0.05 (*), P≤0.01 (**), P≤0.001 (***) and not significant (ns), respectively.

Plants subjected to nights at 4°C and 0°C had significantly lower intercellular CO_2_ concentration (Ci) than those measured in control plants up until 48 h following rewarming, whereas Ci values were higher at 2, 120 and 192 h after the night at −3°C (Figure 1B).

The stomatal conductance (g_s_) increased significantly by 65% and 25%, respectively, 2 and 24 h after the night at 4°C, but it was then reduced by 34% after 48 h (Figure 1C). When cuttings were exposed to 0°C, g_s_ was inhibited by 34% and 59% after 24 and 120 h respectively. In contrast, g_s_ increased at 2, 24 and 192 h following a night at −3°C, with a maximum of 126% at 2 h.

#### Cold night mainly inhibits PSII activity in the flower

**Figure 2:**
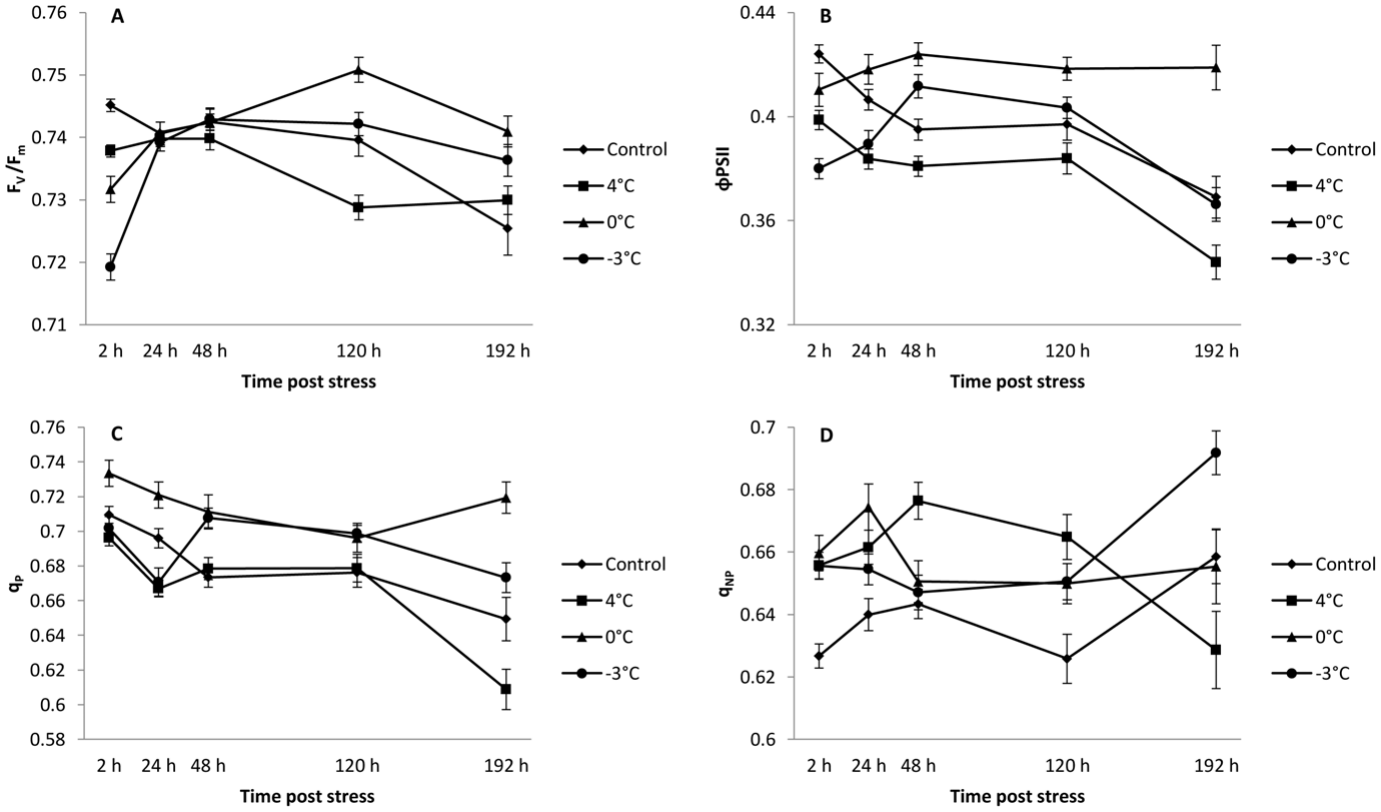
Changes in (A) maximum efficiency of PSII photochemistry after dark adaptation (F_v_/F_m_), (B) effective PSII quantum yield (ΦPSII), (C) photochemical quenching (q_P_) and (D) non-photochemical quenching (q_NP_) in control and 4°C, 0°C or −3°C-treated flowers. Measurements were performed 2, 24, 48, 120 and 192 h after cold treatment. Values are means ± standard errors (n = 45).

**Figure 3:**
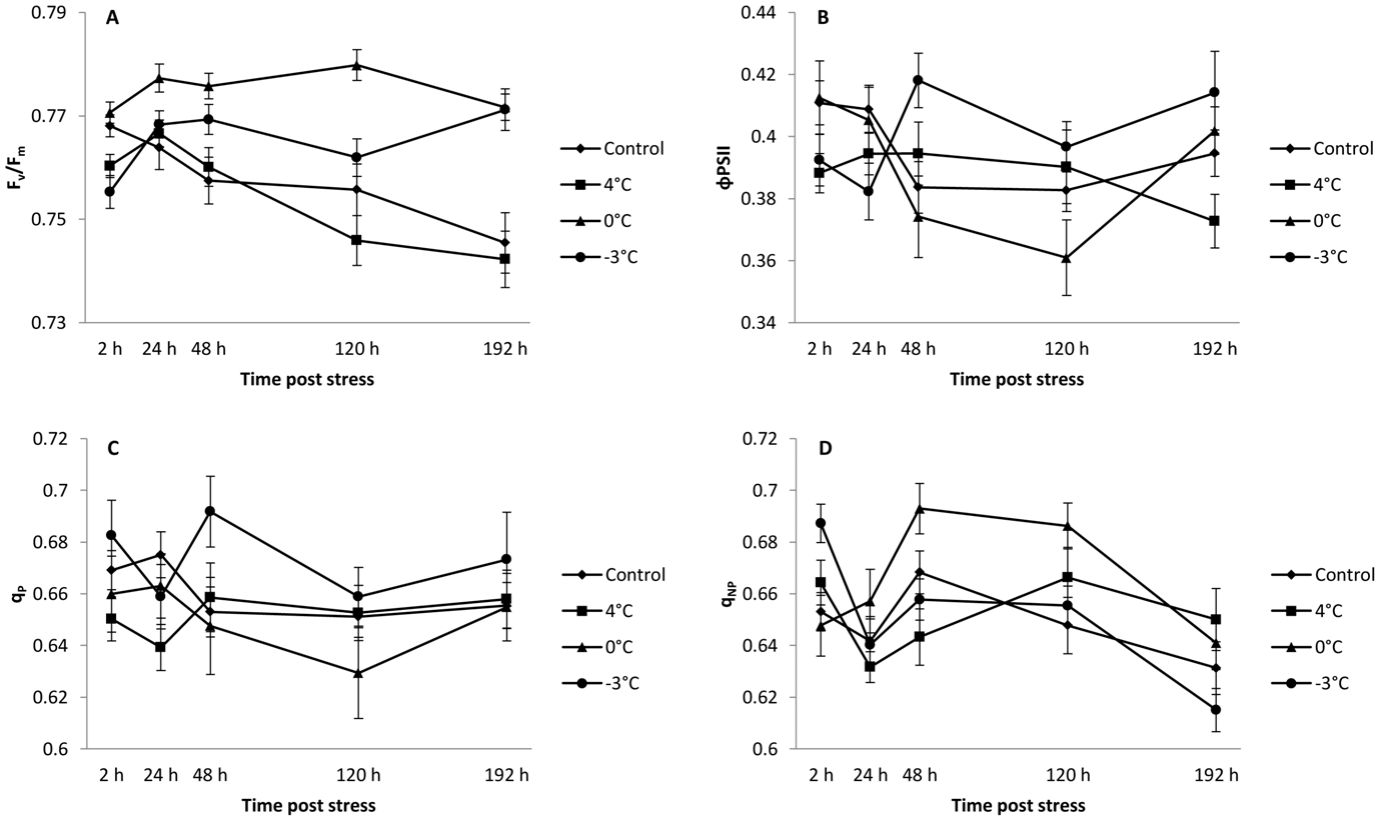
Changes in (A) maximum efficiency of PSII photochemistry after dark adaptation (F_v_/F_m_), (B) effective PSII quantum yield (ΦPSII), (C) photochemical quenching (q_P_) and (D) non-photochemical quenching (q_NP_) in control and 4°C, 0°C or −3°C-treated stalks. Measurements were performed 2, 24, 48, 120 and 192 h after cold treatment. Values are means ± standard errors (n = 45).

**Figure 4:**
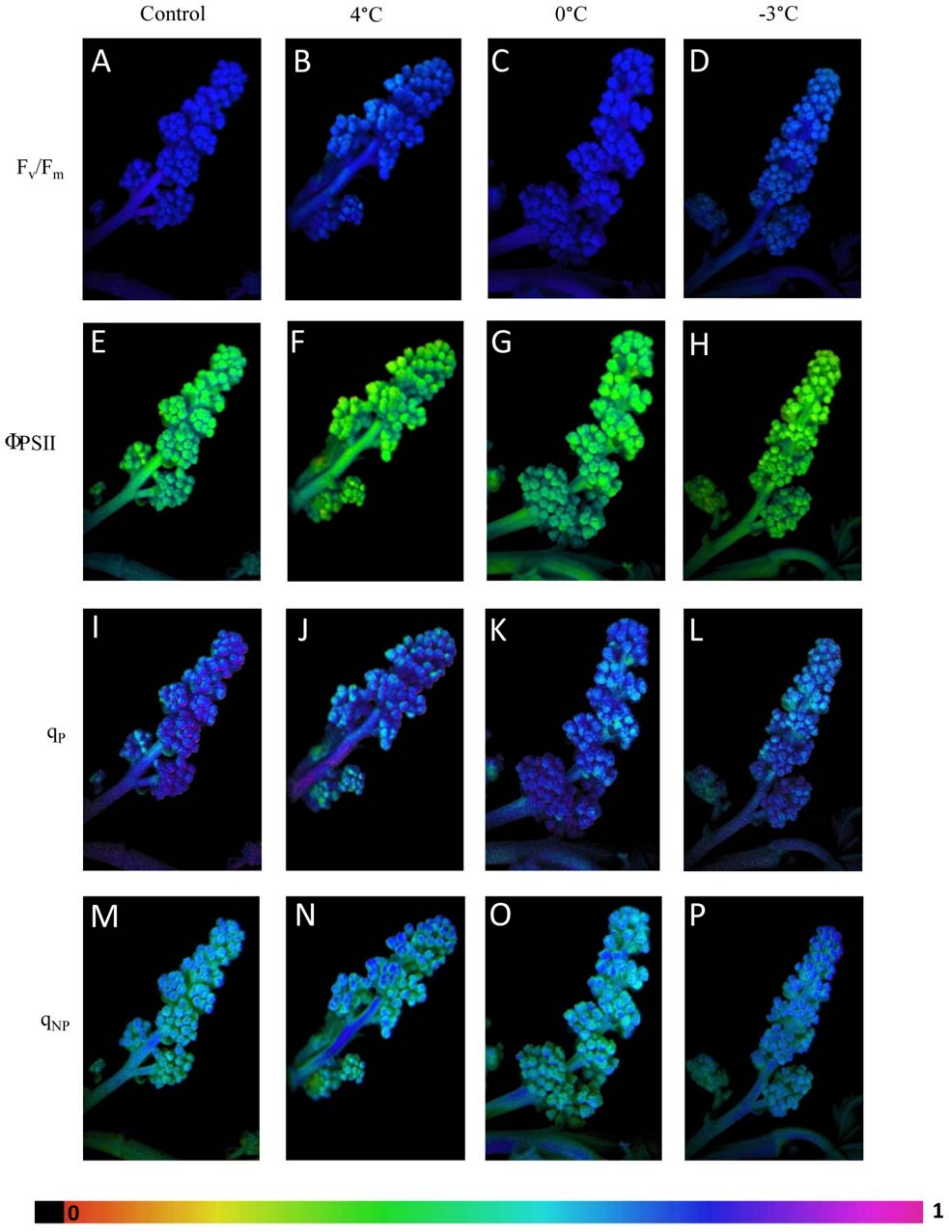
Images of chlorophyll fluorescence parameters at 2 h after the end of the night in control and 4°C, 0°C or −3°C-treated inflorescences.

In vivo chlorophyll fluorescence was used to detect changes in the state of the photosynthetic apparatus [51]. Analysis of chlorophyll a fluorescence clearly indicated that activity of PSII was disturbed in flowers (Figure 2; Table 3) and stalk (Figure 3; Table 3) after each cold night whatever the temperature. In addition, the locations of greatest fluorescence variation within inflorescences were imaged (Figure 4) after 2 h of stress.

**Table 3:** Results of two-way ANOVA of the impact of night temperature on chlorophyll fluorescence parameters.

Variable	Kinetics	Night treatment	Interaction
**Flowers**			
**Fv/Fm**	***	***	***
**φPSII**	***	***	***
**q_P_**	***	***	***
**q_NP_**	ns	***	***
**Stalk**			
**Fv/Fm**	***	***	***
**φPSII**	***	***	***
**q_P_**	ns	*	ns
**q_NP_**	ns	ns	***

Significant results at P≤0.05 (*), P≤0.01 (**), P≤0.001 (***) and not significant (ns), respectively.

In the flowers, the maximum efficiency of PSII (F_v_/F_m_) was most strongly inhibited 2 h after each cold night (−1%, −2% and −3%, respectively, Figures 2A and 4A–D). This parameter was also inhibited at 120 h (−1%) following a night at 4°C, whereas an increase of 2% was recorded after a night at 0°C (Figure 2A). At the end of the experiment, F_v_/F_m_ was no longer inhibited after treatment at 4°C, and it was higher than in the control flowers treated at 0°C (+2%) and −3°C (+2%). In stalks, F_v_/F_m_ was inhibited 2 h (−1%) after a night at 4°C whereas it increased from 24 h (+2%) to 192 h (+3%) after a night at 0°C (Figure 3A). In response to the −3°C stress (Figure 3A), F_v_/F_m_ was also inhibited 2 h after the treatment (−2%) and then increased after 48 h (+2%) and 192 h (+3%).

The PSII quantum yield (ΦPSII) was the parameter most severely inhibited in flowers during the first few hours following cold nights (Figures 2B and 4E–H). Flower ΦPSII was inhibited throughout the period of kinetic measurements after a night at 4°C, with the maximum inhibition (−7%) observed after 192 h. At 0°C, ΦPSII was inhibited after 2 h (−3%) and then increased from 48 h (+7%) to 192 h (+14%). After the −3°C treatment, flower ΦPSII was inhibited at 2 h (10% inhibition) and at 24 h (−4%) but it had recovered by 48 h (+4%). Stalks were less affected than flowers under stress conditions (Figure 3B). Surprisingly, ΦPSII was inhibited by only 5% after 2 h at 4°C, whereas a night at 0°C did not induce any significant difference from the control treatment (Figure 3B). After a night at −3°C, stalk ΦPSII was inhibited by 7% after 24 h and it subsequently recovered, as revealed by an increase after 48 h (+9%) and after 192 h (+5%; Figure 3B).

Photochemical quenching (q_P_) was inhibited after a night at 4°C (Figure 4J), with a maximum inhibition of 6% at 192 h in flowers (Figure 2C). In contrast, after a night at 0°C (Figure 4K), q_P_ increased throughout the period of kinetic measurements, showing a maximum of 11% after 192 h (Figure 2C). Following a night at −3°C, q_P_ was inhibited by 4% after 24 h and then increased by 5% and 3% at 48 and 120 h, respectively (Figures 2C and 4L). In stalks, q_P_ was little affected, except for an inhibition of 5% by 24 h after the night at 4°C and an increase of 6% by 48 h after the night at −3°C (Figure 3C).

Non-photochemical quenching (q_NP_) in flowers generally increased after cold nights (Figures 2D and 4M–P). The maximum increase (+6%) was recorded 120 h after the 4°C treatment. In stalks, q_NP_ was altered after the night treatment as revealed by the one-way ANOVA comparing control and treated inflorescences at 4°C, 0°C and −3°C, respectively (Table 2). It was inhibited 48 h (−4%) after a night at 4°C, whereas it increased by 48 h (+4%) and 120 h (+6%) after a night at 0°C and by 2 h (+5%) after a night at −3°C (Figure 3D).

### Cold night alters the carbohydrate content of inflorescences

Since the main changes in photosynthesis occurred within 2, 24 and 48 h after cold exposure, we restricted analysis of the sugar content in flowers and stalks to the 48 h period following the treatment. We also focused on the main carbohydrates involved in flower nutrition, namely (i) sucrose, since it is the main form in which carbohydrate circulates from the source tissues [52] and also the origin of (ii) glucose and (iii) fructose; and (iv) starch, which is actively mobilized during the development of floral tissues [53], [54] and is the major form of carbohydrate accumulated in grapevine [55].

**Figure 5:**
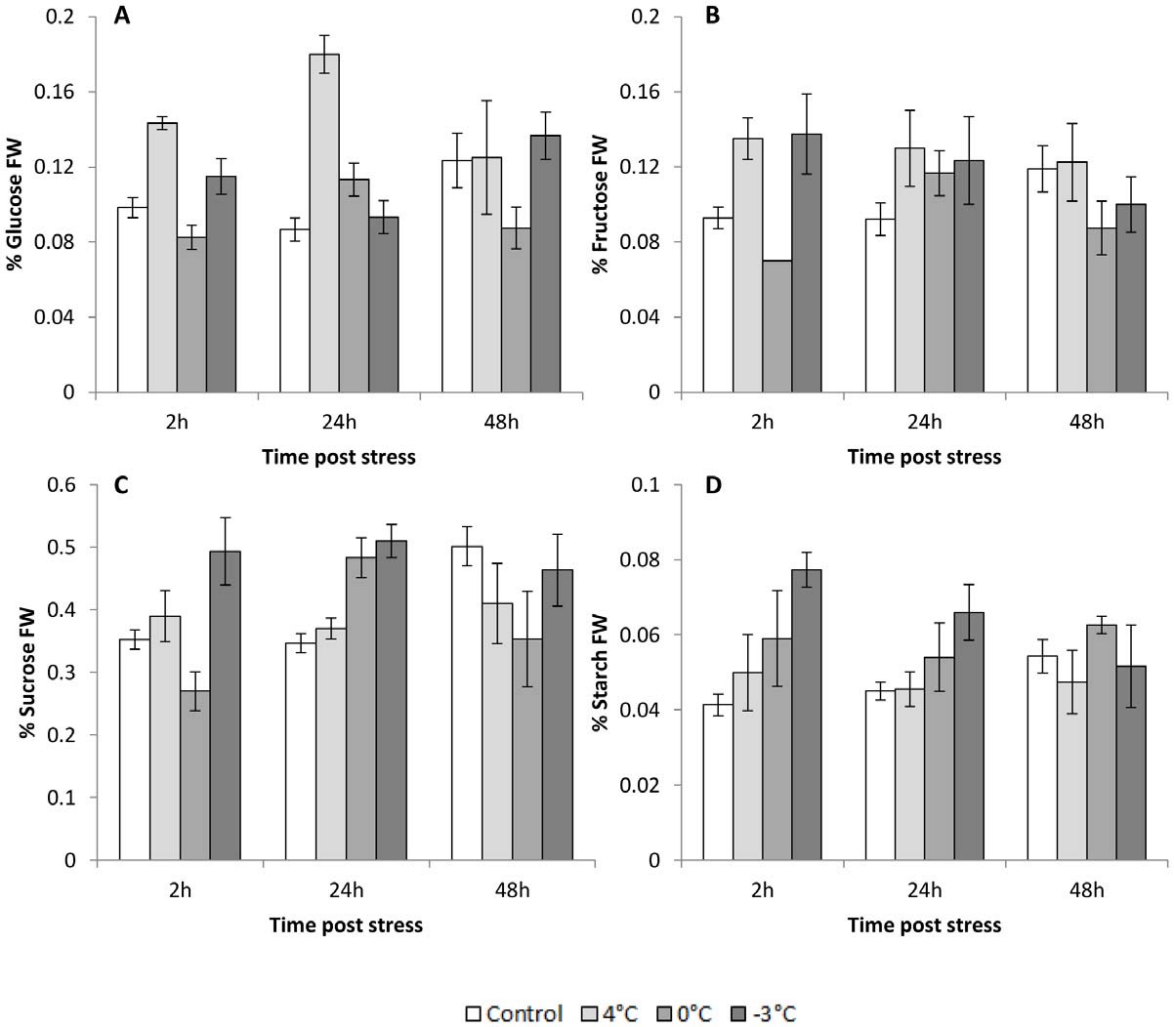
Content, expressed as % fresh weight (FW), of (A) glucose, (B) fructose, (C) sucrose and (D) starch in control and 4°C, 0°C or −3°C-treated inflorescences. Measurements were performed 2, 24 and 48 h after cold treatment. Values are means ± standard errors (n = 27).

Two-way analysis of variance revealed that the night temperature has an impact on carbohydrate contents (Table 4). After a night at 4°C, only hexose concentrations fluctuated. Both glucose and fructose contents were higher in treated plants at 2 h (+46% and +45%, respectively) and 24 h (+107% and +41%, respectively; Figures 5A–B), whereas sucrose and starch contents were stable and similar to those in the control plants (Figures 5C–D).

**Table 4:** Results of two-way ANOVA of the impact of night temperature on carbohydrate content.

Variable	Kinetics	Night treatment	Interaction
**Glucose**	ns	***	**
**Fructose**	ns	***	*
**Sucrose**	*	***	***
**Starch**	ns	***	*

Significant results at P≤0.05 (*), P≤0.01 (**), P≤0.001 (***) and not significant (ns), respectively.

After a night at 0°C (Figure 5), glucose, fructose and sucrose contents were lower than those measured in control inflorescences at 2 h (−15%, −25% and −24%, respectively), but starch content increased (+44%). At 24 h, both glucose and sucrose contents were higher (+30% and +39%, respectively; Figure 5), whereas neither fructose nor starch content was significantly different compared to the control. At 48 h, sucrose content was lower (−30%) in stressed inflorescences.

After a night at −3°C, glucose concentration was stable and similar to that in the control (Figure 5), whereas fructose, sucrose and starch were higher at 2 h (+48%, +40% and +88%, respectively). At 24 h, both sucrose and starch contents were higher compared to the control (+47% for both sugars), whereas fructose content did not fluctuate.

## Discussion

The net photosynthesis in the inflorescence is negative indicating that the amount of carbon fixed by photosynthesis in the inflorescence is less than the amount produced in respiration. A similarly low rate of photosynthesis in grapevine inflorescences was previously reported by Lebon et al. [33], and has also been described in other species such as *Vitis labruscana* [43] and *Malus pumila* [56]. Our results further confirm that inflorescence has a different metabolic profile from that of leaves, in which photosynthesis can reach 13 µmol CO_2_.m^−2^.s^−1^ [19], [57], [58], [59], [60].

Analysis of PSII activity further demonstrates that cold stress differentially affects stalks and flowers, a finding which is in accordance with previous results showing the lower sensitivity of stalks to abiotic stress [61].

### Chilling temperatures at night limit carbon metabolism in grapevine inflorescences via several mechanisms

In grapevine leaves, photosynthesis limitation at chilling temperatures (below 10°C) has already been reported [15], [19], [62], and may be due to stomatal or non-stomatal processes [19]. The results presented here also suggest that photosynthesis in the inflorescence undergoes stomatal or non-stomatal limitations, the extent of which depend on temperature.

#### A cold night at 4°C leads mainly to a non-stomatal limitation of photosynthesis and to hexose accumulation

Within the first 24 h following a night at 4°C, reduced Ci is correlated with higher g_s_ in stressed inflorescences, suggesting a non-stomatal limitation of photosynthesis. Such inhibition in grapevine leaves following a cold night at 5 and 6°C has been shown previously [19], [20], [21], [63] and is likely to be due to (i) a decrease in the rate of PSII activity [20], [63], (ii) effects on photosynthetic pigments and Rubisco activity and (iii) alterations in the abundance of soluble proteins in the PSII reaction center [21].

Fluctuations in gas exchange occur over the same time scale as, and are correlated with, variations in chlorophyll *a* fluorescence, analysis of which clearly revealed that flower PSII activity is impaired since ΦPSII and q_P_ decrease and q_NP_ increases. It thus appears that under cold stress, energy is mainly directed towards heat dissipation rather than being used for CO_2_ fixation.

As reported earlier [52], [64], the loss of PSII activity occurs through (i) photodamage to PSII reaction centers, (ii) down-regulation of electron transport and (iii) photochemical inhibition. This rapid inhibition indicates that PSII is a primary target of cold limitation of the photosynthetic process in grapevine inflorescences.

Accumulation of carbohydrates is one of the most important metabolic adjustments by which plants achieve low temperature tolerance through cold acclimation [6], [65]. In stressed inflorescences of grapevine, the content of both glucose and fructose increases at 2 h and 24 h, which may result from (i) perturbation of mitochondrial respiration, which is known to decrease under chilling [66] and lead to lower glucose consumption by plant cells and/or (ii) carbohydrate import from other plant organs. The increase in glucose and fructose concentrations in cold-stressed inflorescences is probably correlated with import of sucrose, which is the major carbohydrate transport form in grapevine [58].

#### Cold night at 0°C leads mainly to stomatal limitation of photosynthesis and to carbohydrate accumulation

Net photosynthesis is disturbed 2 h after the end of the night at 0°C, indicating that this temperature has a different effect on inflorescence metabolism than a night at 4°C. Soon after the end of the night, Pn is higher in stressed inflorescences while Ci is lower, strongly suggesting stomatal limitation of photosynthesis. PSII activity is inhibited 2 h after the chilling treatment, with a strong inhibition of the ΦPSII and F_v_/F_m_ parameters as well as a higher q_NP_. However, a night at 0°C leads to a higher q_P_ in stressed inflorescences. Taken together, these results suggest an increase in the proportion of open PSII reaction centers, which indicates that PSII photochemical capacity is slightly altered following a night at 0°C. Subsequently, reduced Pn is correlated with lower Ci and g_s_ in stressed inflorescences, which confirms the likelihood of stomatal photosynthetic limitation. Stomatal limitation in leaves following a cold night is a common symptom in many chilling sensitive species, as has been previously shown in coffee [67], cacao [68], tomato [69], and mango [70]. Decreased stomatal conductance following cold exposure has also been shown in soybean, although in some cultivars, mesophyll limitation dominated the inhibition of CO_2_ assimilation [71]. In grapevine leaves, although stomatal effects can be detected after a cold night (5°C), non-stomatal effects predominate [15].

Moderate cold stress (0°C) seems to make inflorescences mobilize soluble sugars but not starch. A cold night is followed by a simultaneous decrease of soluble sugars (glucose, fructose and sucrose) and starch accumulation as previously shown [28]. Combining this result with those of gas exchange analysis suggests that respiration is drastically impaired by a night at 0°C [66]. Starch accumulation may be due to storage of carbohydrates taken up by other plant organs, especially after the end of the cold night. One day after cold exposure, inflorescence metabolism seems to undergo modifications, showing an accumulation of glucose and sucrose at 24 h without variation in starch content.

### Freezing night-time temperature severely impairs photosynthesis in the inflorescence and induces accumulation of both soluble and insoluble carbohydrate

One night at −3°C has a drastic impact on inflorescence Pn, indicating that Ci and g_s_ are higher in stressed plants; this occurs concomitantly with PSII inhibition, the end result being a non-stomatal limitation of photosynthesis.

Inhibition of the Calvin cycle, for example through loss of Rubisco activity, might explain the increase of Ci and could contribute to a reduction in Pn [16]. Environmental stresses such as cold exposure may also decrease the rate of photosynthesis because of changes in the rate of diffusion of CO_2_ from the atmosphere to the carboxylation site [72], [73]. Nevertheless, the marked increase of g_s_ observed in the present study suggests a deregulation of stomatal function following the freezing night. Previous studies have shown that the cell membranes are the primary sites of freezing injury in plants [74], [75], which is consistent with disturbance of stomatal functioning. Our results thus suggest that the limitations of inflorescence carbon metabolism after a freezing night are not only due to stomatal/non-stomatal factors. This inference is in agreement with an observed decrease in the photosynthetic response in grape leaves exposed to heat stress, which was attributed mainly to non-stomatal factors but which was also associated with a stomatal response [76].

Carbohydrate analysis showed that starch strongly accumulates after a freezing night. Fructose and sucrose contents are also higher in stressed inflorescences, in spite of the strong inhibition of PSII, suggesting a major disturbance of respiration and/or significant carbohydrate import into stressed inflorescences within the first 24 h following rewarming.

### Conclusions

Grapevine inflorescence metabolism is impaired after a cold night because of disturbed gas exchange and loss of PSII activity. In particular, ΦPSII appears to be the most strongly inhibited parameter, suggesting its potential as an early marker for stress in inflorescences. The PSII inhibition is accompanied by an increase in q_NP_ which is the most common form of protection against photon excess in higher plants [77], limiting the production of harmful oxygen species [78] and affording photoprotection to the photosynthetic apparatus [79]. The inflorescences of grapevine submitted to cold temperatures may respond in the same way.

Disruption of inflorescence physiology is due mainly to non-stomatal photosynthetic limitations after a night at 4°C, whereas it is due mainly to stomatal photosynthetic limitations at 0°C. The freezing night (−3°C) seems to have a more drastic impact than the chilling treatment, deregulating stomatal function and inhibiting PSII activity. Our results further suggest that grapevine inflorescences modify their metabolism by importing carbohydrates, as shown by the high level of soluble carbohydrates despite limitation of the photosynthetic process. Physiological inhibition combined with carbohydrate import into the inflorescence may lead to an imbalance between carbon source and sink at the whole plant level and consequently to impaired development of reproductive structures, resulting in flower abortion.
